# A reappraisal of the phylogeny and historical biogeography of *Sparganium* (Typhaceae) using complete chloroplast genomes

**DOI:** 10.1186/s12870-022-03981-3

**Published:** 2022-12-15

**Authors:** Qiaoyu Zhang, Eugeny A. Belyakov, Alexander G. Lapirov, Yixuan Zhao, Joanna Freeland, Xinwei Xu

**Affiliations:** 1 National Field Station of Freshwater Ecosystem of Liangzi Lake, College of Life Sciences, Wuhan University, Wuhan, People’s Republic of China; 2 Papanin Institute for Biology of Inland Waters Russian Academy of Sciences, Borok, Nekouz District, Yaroslavl Region, 152742 Russia; 3 Department of Biology, Trent University, Peterborough, ON Canada

**Keywords:** *Sparganium*, Chloroplast genome, Phylogeny, Divergence time, Ancestral area

## Abstract

**Background:**

*Sparganium* (Typhaceae) is a widespread temperate genus of ecologically important aquatic plants. Previous reconstructions of the phylogenetic relationships among *Sparganium* species are incompletely resolved partly because they were based on molecular markers comprising < 7,000 bp. Here, we sequenced and assembled the complete chloroplast genomes from 19 *Sparganium* samples representing 15 putative species and three putative subspecies in order to explore chloroplast genome evolution in this genus*,* clarify taxonomic lineages, estimate the divergence times of *Sparganium* species, and reconstruct aspects of the biogeographic history of the genus.

**Results:**

The 19 chloroplast genomes shared a conserved genome structure, gene content, and gene order. Our phylogenomic analysis presented a well-resolved phylogeny with robust support for most clades. Non-monophyly was revealed in three species: *S. erectum*, *S. eurycarpum*, and *S. stoloniferum*. Divergence time estimates suggest that the two subgenera of *Sparganium* split from each other ca. 30.67 Ma in the middle Oligocene. The subgenus *Xanthosparganium* diversified in the late Oligocene and Miocene, while the subgenus *Sparganium* diversified in the late Pliocene and Pleistocene. Ancestral area reconstruction suggested that the two subgenera may have originated in East Eurasia and North America.

**Conclusion:**

The non-monophyletic nature of three putative species underscores the necessity of taxonomic revision for *Sparganium*: *S. stoloniferum* subsp. *choui* may be more appropriately identified as *S. choui,* and subspecies of *S. erectum* may be in fact distinct species. The estimated diversification times of the two subgenera correspond to their species and nucleotide diversities. The likely ancestral area for most of subgenus *Xanthosparganium* was East Eurasia and North America from where it dispersed into West Eurasia and Australia. Most of subgenus *Sparganium* likely originated in North America and then dispersed into Eurasia. Our study demonstrates some of the ways in which complete chloroplast genome sequences can provide new insights into the evolution, phylogeny, and biogeography of the genus *Sparganium*.

**Supplementary Information:**

The online version contains supplementary material available at 10.1186/s12870-022-03981-3.

## Background

*Sparganium* L. is an aquatic perennial genus comprising approximately 14–19 species [1–4]. It mainly occurs in temperate and cool regions of the Northern Hemisphere with only two species extending from eastern Asia southward into Australia and/or New Zealand [1, 2]. *Sparganium* species often dominate wetlands and play important ecological roles in aquatic communities [1, 2]. The tuberous rhizome of *S*. *stoloniferum* is widely used as a gynecological drug in traditional Chinese medicine [5].

Species delimitation is difficult in *Sparganium* due to phenotypic plasticity and interspecific hybridization. The comprehensive *Sparganium* monographs by Cook and Nicholls [1, 2] recognized 14 species and six subspecies, divided between two subgenera: Subgenus *Xanthosparganium* included seven species and one subspecies with translucent perianth segments. Subgenus *Sparganium* contained seven species and five subspecies with dark brown to black perianth segments [1, 2]. Sulman et al. [6] realigned the subgenera to conform to two clades in a phylogeny of *Sparganium* that was based on two chloroplast DNA fragments and two nuclear genes. The revised subgenus *Sparganium* included *S. erectum* and *S. eurycarpum*, both of which have bilocular ovary and endocarps with longitudinal ridges. The revised subgenus *Xanthosparganium* included the remaining 12 species, all of which have unilocular ovary and endocarps without longitudinal ridges [1, 2, 6]. Cook and Nicholls [1] treated *S. acaule* as a subspecies (*S. emersum* subsp. *acaule*), but it was resurrected as a species by Ito et al. [7] due to the non-monophyletic nature of *S. emersum* sensu lato in a phylogeny of *Sparganium* that was based on six cpDNA regions plus one nuclear gene. Nevertheless, the phylogenetic relationships among *Sparganium* species remain incompletely resolved, partly because they have so far been based on limited genetic data (< 4,000 bp [6] or < 7,000 bp [7]).

*Sparganium* is an early-diverging lineage in Poales [8] with abundant fossils from the Paleocene [6, 9, 10]. These fossils show that the ancestral species from the two subgenera were distinct by the Oligocene, and by the Miocene the endocarps of described fossil species are very difficult to distinguish from extant species [1, 11]. However, previous divergence time analyses using molecular dating based on DNA sequences of four gene regions suggested a mid-Miocene crown origin and Pliocene diversification of *Sparganium* [6], which is inconsistent with the fossil record. Therefore, it is necessary to re-estimate the divergence time of *Sparganium* using a more extensive molecular genetic dataset.

In plants, no universal barcode consistently discriminates among plant species and reveals phylogenetic relationships. Researchers are therefore increasingly generating whole-plastid genome sequences to study taxonomy and biogeography [12, 13]. For example, complete chloroplast genome sequences have provided higher resolutions of phylogenetic relationships among plants compared to phylogenetic reconstructions that were based on just a few chloroplast fragments [13–15]. Here, we sequenced and assembled chloroplast genomes of 19 *Sparganium* samples, which were provisionally identified as 15 species and three subspecies. Our aims were to 1) investigate chloroplast genome evolution in *Sparganium*; 2) clarify the evolutionary relationships among *Sparganium* species; and 3) estimate the divergence times and infer the ancestral areas of *Sparganium*.

## Results

### Feature of chloroplast genomes

**Fig. 1:**
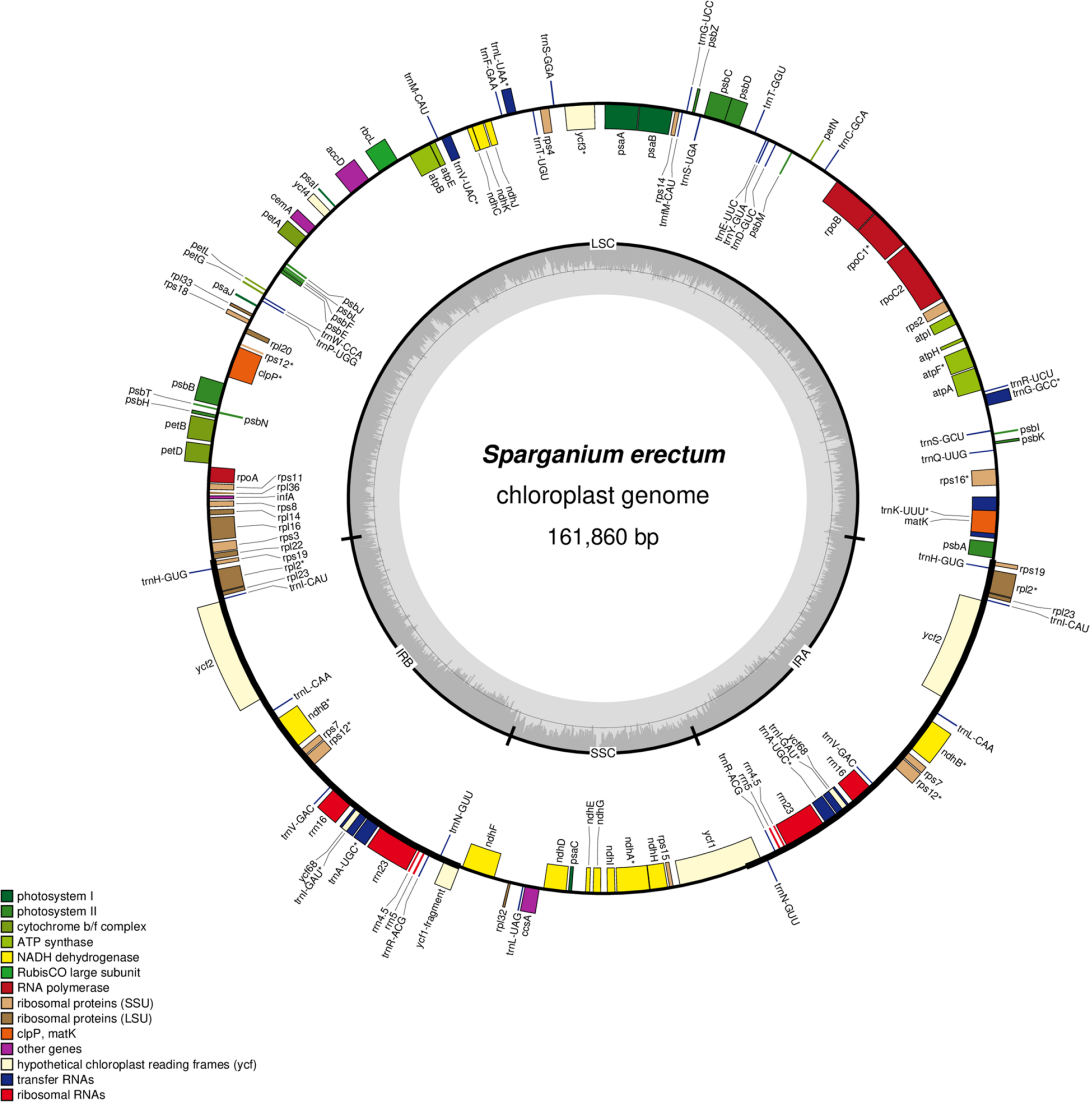
The chloroplast genome maps of *Sparganium* species

The chloroplast genomes of all *Sparganium* samples were successfully assembled from genome skimming data. Their sizes ranged from 161,487 to 162,331 bp with a typical quadripartite structure (Fig. 1), including two Inverted Repeat (IR) regions (26,882–27,037 bp), one Large Single Copy (LSC) region (88,026–89,281 bp) and one Small Single Copy (SSC) region (18,684–19,120 bp) (Table 1). Each of the 19 chloroplast genomes that we reconstructed encoded 114 unique genes comprising 80 protein coding genes (PCGs), 30 tRNA genes, and four rRNA genes. The gene arrangement was identical in each genome. The overall GC content of the genomes (36.7–36.9%) was conserved across species (Table 1).

**Table 1:** Detailed information of chloroplast genomes of *Sparganium* species

*Sparganium* species	Location	Latitude/ Longitude	Collection Number	Genome size (bp)	LSC length (bp)	SSC length (bp)	IR length (bp)	Number of unique genes (PCGs, rRNAs, rRNAs)	GC content (%)	GenBank accession number
*S. acaule*	Ontario, Canada	48.21 N/82.28 W	XUNA026	161,820	88,824	18,976	27,010	114 (80, 30, 4)	36.8	MW829767
*S. androcladum*	Ontario, Canada	46.96 N/79.79 W	XUNA033	162,032	89,025	18,933	27,037	114 (80, 30, 4)	36.8	MW789349
*S. angustifolium*	Murmansk, Russia	69.22 N/29.31 E	E19	161,830	89,097	18,735	26,999	114 (80, 30, 4)	36.9	MW810982
*S. emersum*	Yaroslavl, Russia	56.82 N/39.36 E	RU116	161,760	89,002	18,750	27,004	114 (80, 30, 4)	36.9	MW810983
*S. erectum*	Ryazan, Russia	54.39 N/40.98 E	AF28	161,860	88,958	19,120	26,891	114 (80, 30, 4)	36.8	MW810984
*S. erectum* subsp. *microcarpum*	Yaroslavl, Russia	57.54 N/38.04 E	E86	161,792	88,962	19,030	26,900	114 (80, 30, 4)	36.8	MW817016
*S. erectum* subsp. *neglectum*	Krasnodar, Russia	44.44 N/38.19 E	be13	161,810	88,964	19,034	26,906	114 (80, 30, 4)	36.8	MW817017
*S. eurycarpum*_NS	Nova Scotia, Canada	45.08 N/64.49 W	Tisshaw s.n	161,812	88,935	19,049	26,914	114 (80, 30, 4)	36.9	MW829761
*S. eurycarpum*_ON	Ontario, Canada	44.93 N/76.64 W	XUAN042	161,828	89,025	19,029	26,887	114 (80, 30, 4)	36.8	MW829768
*S. fallax*	Fujian, China	26.81 N/116.89 E	Xu3372	161,838	89,042	18,774	27,011	114 (80, 30, 4)	36.8	MW874418
*S. fluctuans*	Ontario, Canada	49.25 N/91.11 W	XUNA052	162,191	89,065	19,092	27,017	114 (80, 30, 4)	36.8	MW817018
*S. glomeratum*	Yaroslavl, Russia	58.74 N/38.75 E	E72	161,487	88,777	18,700	27,005	114 (80, 30, 4)	36.9	MW817014
*S. gramineum*	Yaroslavl, Russia	56.82 N/39.36 E	Ru114	162,053	89,053	18,978	27,011	114 (80, 30, 4)	36.8	MW817019
*S. hyperboreum*	Arkhangelsk, Russia	64.68 N/43.74 E	E27	162,331	89,281	19,068	26,991	114 (80, 30, 4)	36.7	MW817015
*S. japonicum*	Heilongjiang, China	47.12 N/129.22 E	Xu3964	161,968	88,996	18,978	26,997	114 (80, 30, 4)	36.8	MW829762
*S. natans*	Tver, Russia	54.82 N/26.86 E	E17	161,635	88,842	18,821	26,986	114 (80, 30, 4)	36.8	MW829763
*S. stoloniferum*	Xinjiang, China	41.93 N/86.69 E	Xu4350	161,766	88,965	19,037	26,882	114 (80, 30, 4)	36.9	MW829764
*S. stoloniferum* subsp. *choui*	Inner Mongolia, China	48.13 N/123.46 E	Xu568	161,865	88,953	19,098	26,907	114 (80, 30, 4)	36.8	MW829765
*S. subglobosum*	Heilongjiang, China	47.83 N/133.69 E	Xu249	161,732	89,052	18,686	26,997	114 (80, 30, 4)	36.9	MW829766

### Comparison of border regions and sequence identity

**Fig. 2:**
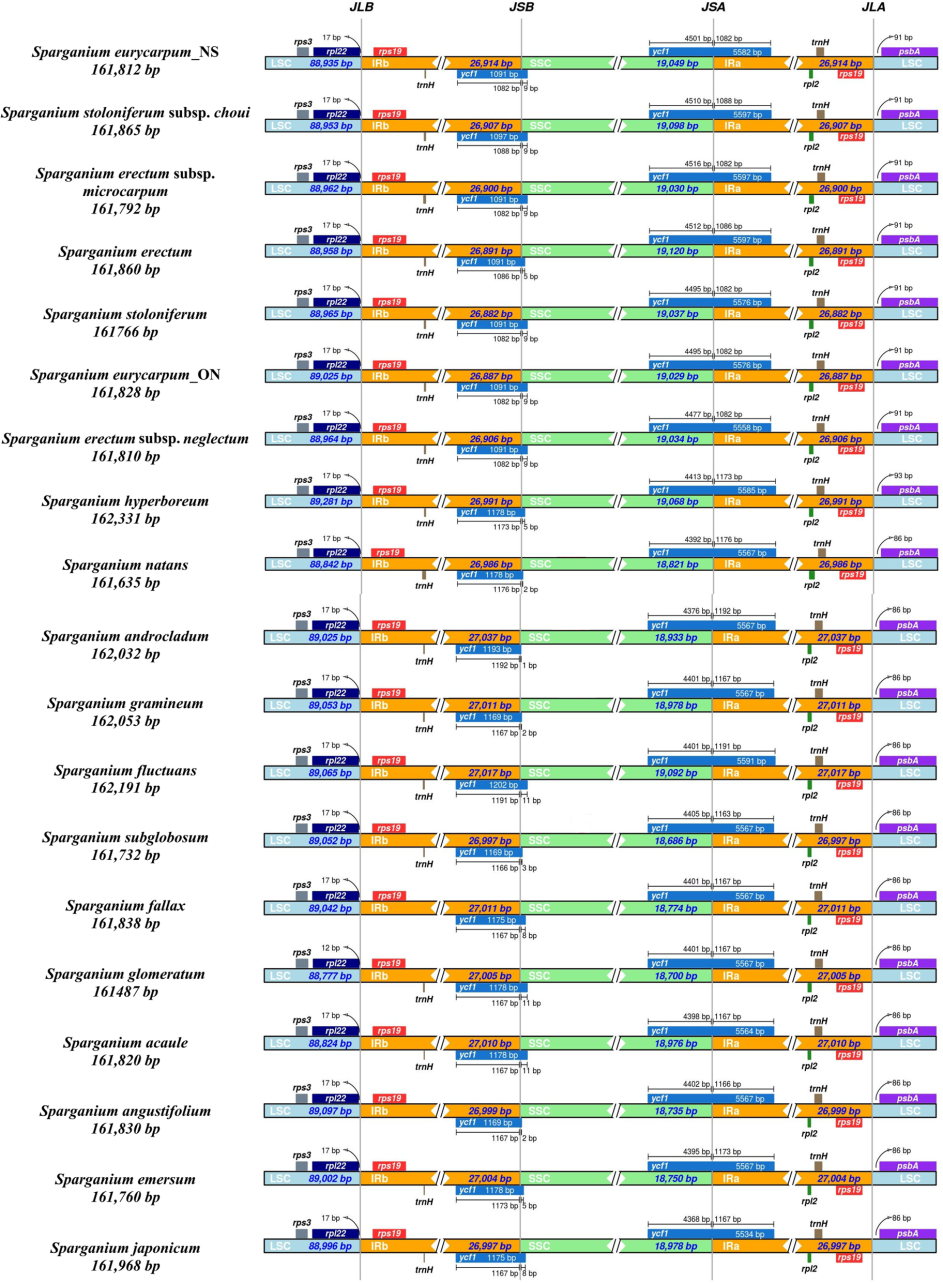
Comparison of the boundary of chloroplast genomes of *Sparganium* species

Potential IR expansion or contraction was assessed by comparing the LSC/IR and SSC/IR junctions across species. The locations of LSC/IRa (JLA) and LSC/IRb (JLB) junctions were the same for all *Sparganium* species: the JLA was consistently located at the *psbA-rps19* intergenic spacer (IGS), and the JLB was consistently located at the *rpl22-rps19* intergenic spacer (Fig. 2). The distance between *psbA* and JLA was 91 bp for all species of subgenus *Sparganium*, and 86 bp for all species of subgenus *Xanthosparganium* except *S. hyperboreum* (93 bp). The distance between *rpl22* and JLB was 17 bp for all species except *S. glomeratum* (12 bp). The locations of SSC/IRa (JSA) and SSC/IRb (JSB) were also conserved across species, with the IR region consistently expanding into the *ycf1* gene at the JSA or JSB junctions (Fig. 2).

The sequence identity analysis revealed a high similarity (97.53–99.6%) among chloroplast genomes of *Sparganium* species, especially for species within the subgenus *Sparganium* that were invariant at > 99% of nucleotide sequences (Table S1). The LSC and SSC regions were more divergent than the IR region (Figs. S1 and S2). In addition, four IGS regions (*trnS-trnG*, *ndhF-rpl32, accD-psaI* and *petA-psbJ*), and two PCGs (*ndhF* and *ndhE*) showed relatively high nucleotide diversity with Pi values greater than 0.02 (Fig. S2), making these candidate molecular markers for future phylogenetic and phylogeographic studies.

### Phylogenetic analysis

**Fig. 3:**
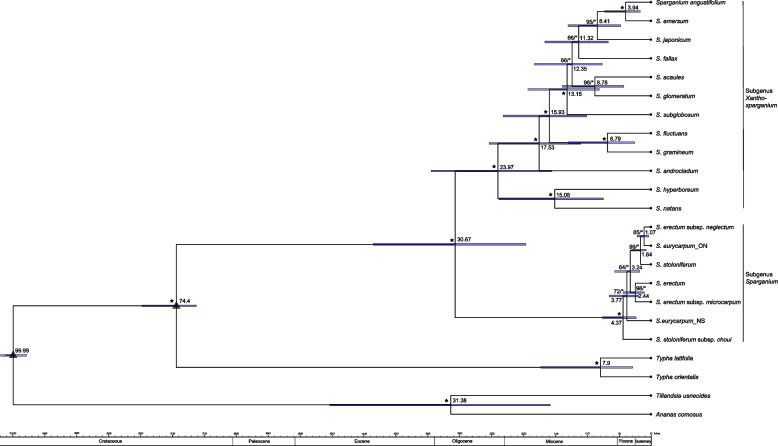
Phylogenomic analysis and divergence time dating of *Sparganium*. The phylogenetic tree was reconstructed from sequences of 80 protein coding genes. Asterisks indicate bootstrap support = 100/posterior probability = 1.00. Triangles indicate the fossil calibration nodes and numbers close to nodes refer to the mean divergent time estimates. Blue bars indicate 95% highest posterior distributions

The aligned length of 80 PCGs was 70,612 bp with 3,507 informative sites. Identical topologies were revealed using the maximum likelihood (ML) and Bayesian inference (BI) methods (Fig. 3). *Sparganium* comprises a strongly supported monophyletic group (BS = 100, PP = 1) divided into two unambiguous clades corresponding to subgenus *Sparganium* (BS = 100, PP = 1) and subgenus *Xanthosparganium* (BS = 100, PP = 1). None of the three species in the subgenus *Sparganium* were monophyletic. *Sparganium stoloniferum* subsp. *choui* was placed as sister to the remaining species or subspecies, which formed a clade with high support (BS = 72, PP = 1). Two strongly supported clades, *S. stoloniferum* + *S. erectum* subsp. *neglectum* + *S. eurycarpum*_ON (BS = 99, PP = 1) and *S. erectum* subsp. *microcarpum* + *S. erectum* (BS = 98, PP = 1), were clustered with high support (BS = 64, PP = 1) and resolved as the sister group of *S. eurycarpum*_NS. In the subgenus *Xanthosparganium*, four sister species pairs were revealed, and the backbone was well resolved with robust support for most of the nodes. The topology showed a hierarchical branching structure and the root branching order was *S. natans* + *S. hyperboreum*, *S. androcladum*, *S. gramineum* + *S. fluctuans*, *S. subglobosum*, *S. glomeratum* + *S. acaule*, *S. fallax*, *S. japonicum*, and *S. emersum* + *S. angustifolium*.

### Divergence time estimation and ancestral area reconstruction

The stem age of *Sparganium* was estimated to be 74.4 Ma (95% highest posterior densities (HPD):71.28–79.81 Ma). Two subgenera split from each other at approximately 30.67 Ma (95% HPD: 19.58–43.52 Ma) in the middle Oligocene (Fig. 3). The subgenus *Xanthosparganium* began to diversify approximately 23.97 Ma (95% HPD: 15.54–34.45 Ma) in the late Oligocene, with the majority of species diversification occurring in the Miocene other than the divergence between *S. emersum* and *S. angustifolium*, which did not occur until the Pliocene. The subgenus *Sparganium* did not differentiate until approximately 4.37 Ma (95% HPD: 2.3–7.58 Ma) in the Pliocene, and most of the species/subspecies divergence within this subgenus *Sparganium* occurred during the Pleistocene.

**Fig. 4:**
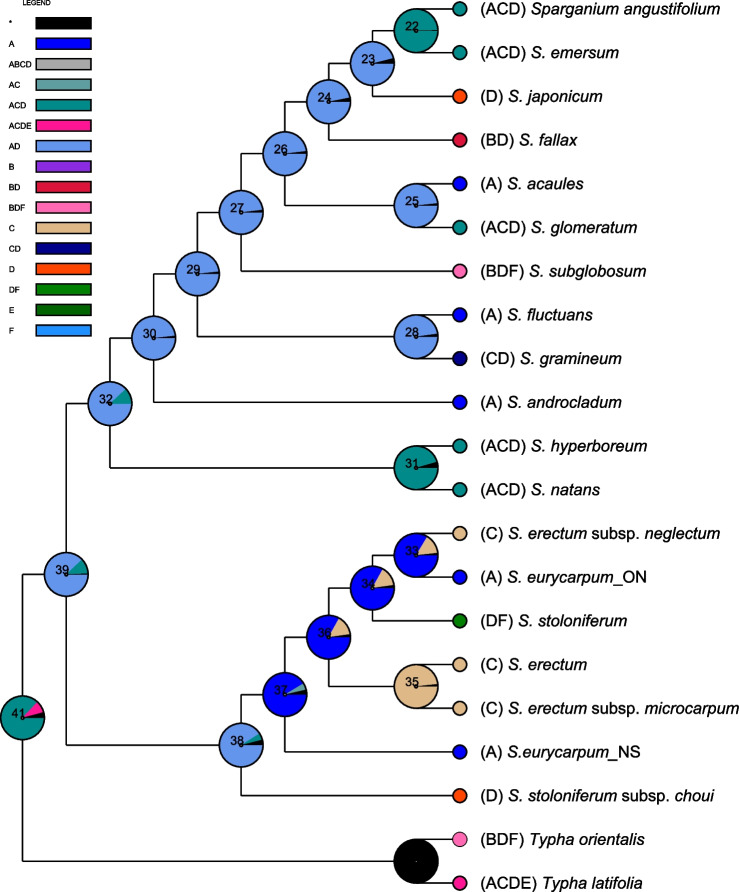
Reconstruction of the most likely ancestral areas of *Sparganium*. The pie charts at each node were obtained using the BioGeoBEARS analysis. Letters represent the following biogeographic regions: (**A**) North America, (**B**) Indo-Pacific, (**C**) West Eurasia, (**D**) East Eurasia, (**E**) Africa, and (**F**) Australia

Based on BioGeoBEARS analysis, the BAYAREALIKE model was supported as the best-fit model (AICc_wt = 0.79) for ancestral area reconstruction. East Eurasia and North America were suggested as high probability ancestral areas for the genus and the two subgenera (Fig. 4). In subgenus *Xanthosparganium*, the ancestral area of all nodes was East Eurasia and North America except for nodes 22 (*S. emersum* + *S. angustifolium*) and 31 (*S. natans* + *S. hyperboreum*), which included West Eurasia as a likely ancestral area. In subgenus *Sparganium*, the ancestral area of all nodes was North America except for node 35 (*S. erectum* subsp. *microcarpum* + *S. erectum*), for which West Eurasia was identified as the most likely ancestral area. Expansion from ancestral areas involved 30 dispersal events across 15 nodes (all except 35, 37, and 38) along with five vicariance events across five nodes (28, 33, 34, 36, and 38; Table S2).

## Discussion

### Chloroplast genome evolution

The evolution of chloroplast genomes often entails gene inversions, translocations, losses or rearrangements [16, 17]. However, the 19 *Sparganium* chloroplast genomes were highly conserved, with each having the same numbers and arrangements of genes. Expansion or contraction of the IR is common in chloroplast genomes and plays an important role in the size variation of chloroplast genomes in angiosperms [18]. Among *Sparganium* chloroplast genomes, the lengths of the IR region were comparable (26,882 bp-27,037 bp), as were the locations of JLA, JLB, JSA and JSB, thus further illustrating the conservative nature of *Sparganium* chloroplast genomes.

Four chloroplast DNA genes (*matK*, *rbcL*, *rpoB* and *rpoC1*) and four IGS regions (*trnL*-*trnF*, *petA-psbJ*, *psbM*-*trnD* and *trnC*-*petN*) were used in two previous studies on the phylogeny of *Sparganium* [6, 7]. No full resolution of phylogenetic reconstruction was achieved due to the low nucleotide diversity of these regions (except for *petA-psbJ*, Fig. S2). The relatively variable regions identified in this study (Fig. S2), including *trnS-trnG*, *ndhF-rpl32, accD-psaI*, *petA-psbJ*, *ndhF* and *ndhE*, should provide more informative molecular markers for future phylogenetic or phylogeographic studies in this genus.

### Phylogenetic relationship

Our phylogenomic analysis revealed two strongly supported clades, corresponding to the subgenera *Xanthosparganium* and *Sparganium* that were proposed in a previous study based on partial cpDNA and nuclear sequences plus stigma and endocarp features [6]. Members of the subgenus *Xanthosparganium* species are floating or emergent and have unilocular ovary and endocarps without longitudinal ridges [1, 6]. The membership that we identified for this subgenus agrees with that identified in earlier studies [6, 7], however, our phylogenetic reconstruction had no polytomies and high support for most clades (Fig. 3), and thus more clearly elucidates the relationships among the 12 species within this subgenus (Fig. 3). Four previously identified sister species pairs, *S. natans* + *S. hyperboreum*, *S. gramineum* + *S. fluctuans* [6], *S. glomeratum* + *S. acaule*, and *S. emersum* + *S. angustifolium* [7], were confirmed by our phylogenomic analysis with 96–100% support. The morphological similarities between *S. natans* + *S. hyperboreum* and between *S. gramineum* + *S. fluctuans* have been described by Cook and Nicolls [1]. *Sparganium glomeratum* + *S. acaule* share several features including congested female heads, 1–3 male heads, and the lowest inflorescence bract that is longer than the flowering stem [7]. *Sparganium emersum* + *S. angustifolium* differ in leaf shape and male heads [1] although they are often confused when *S. emersum* is in its floating form; others have suggested that these be considered a single, complex species [19, 20].

Species within the subgenus *Sparganium* species are emergent and have bilocular ovary and endocarps with longitudinal ridges [1, 6]. There has been some debate about taxonomic demarcations in this subgenus, which in our study included taxa that we initially identified as *S. stoloniferum*, *S. stoloniferum* subsp. *choui*, *S. eurycarpum* (one sample from Ontario, Canada and one sample from Nova Scotia, Canada), *S. erectum*, *S. erectum* subsp. *neglectum*, and *S. erectum* subsp. *microcarpum*. *Sparganium stoloniferum* and *S. stoloniferum* subsp. *choui* did not form a monophyletic group (Fig. 3). Subspecies *S. stoloniferum* subsp. *choui* is sometimes considered to be a distinct species, *S. choui* [3, 21], because compared with *S. stoloniferum* it has a short panicle, only one male head per branch, a short anther length, and a small fruit [21]. The non-monophyly of *S. stoloniferum* revealed by our whole-genome phylogeny, combined with morphological differences, suggest that *S. choui* may be more appropriately considered a species than a subspecies. *Sparganium eurycarpum* was also polyphyletic, and the sample from Ontario groups with *S. erectum* subsp. *neglectum* have high support. Related to this, four genetic groups identified on the basis of AFLPs, genome sizes, and fruit morphology, corresponded to four subspecies of *S. erectum* (subsp. *erectum*, subsp. *microcarpum*, subsp. *neglectum* and subsp. *oocarpum*) that were distributed across 64 populations in the Czech Republic [2, 22]. In addition to their non-monophyly in phylogenetic tree (Fig. 3), it may therefore be more appropriate to assign species-status to the subspecies of *S. erectum*. However, the recency with which subgenus *Sparganium* diversified, and the associated low levels of sequence divergence, mean that further investigation is needed to clarify some of the taxonomic groups within subgenus *Sparganium.* Future studies should also compare phylogenies based on nuclear genes with those based on whole chloroplast genomes to test the possibility that historical and/or more recent hybridization, which has been reported among multiple *Sparganium* species and subspecies [1; 7; 20], is obscuring the phylogenetic inferences in this subgenus.

### Biogeographical reconstruction

Accurate estimates of divergence times are needed before we can fully understand biogeographic histories. The stem age of *Sparganium* or the crown age of Typhaceae was estimated to be 74.4 Ma (Fig. 3), which is similar to the previously reported age of 72 Ma [6] and agrees with the earliest known fossils of *Typha* from the late Cretaceous [23]. Our time-calibrated tree indicated a crown age of *Sparganium* at 30.67 Ma, which is much older than the 13 Ma reported in Sulman et al. [6] although the two studies used the same calibration points. However, Sulman et al. [6] based their estimate on < 4,000 bp, and divergence estimates should be more reliable when based on whole chloroplast genomes. The crown age of *Sparganium* in the Oligocene and its main diversification in the Miocene (Fig. 3) are also consistent with the finding that the endocarps of fossil species from the Miocene onwards are very difficult to distinguish from extant species [1]. The subgenus *Xanthosparganium* began to diversify in the late Oligocene (an estimated 24 Ma) while the subgenus *Sparganium* did not begin to differentiate until the late Pliocene (an estimated 4.34 Ma). These dates are consistent with the species diversity of the two subgenera: the subgenus *Xanthosparganium* contains many species with diverse life forms including boreal and temperate species as well as emerged and floating-leaved species. In contrast, the subgenus *Sparganium* contains fewer species, but all of which are robust, erect, and temperature species with similar morphological characteristics [1, 2]. In addition, as noted earlier, nucleotide diversity across the chloroplast genome was lower in subgenus *Sparganium* than in subgenus *Xanthosparganium.*

The BioGeoBEARS analysis suggested East Eurasia and North America as the ancestral areas for *Sparganium* and its two subgenera (Fig. 4), which is consistent with the abundant *Sparganium* fossil records from these regions [1, 2, 6]. In the subgenus *Xanthosparganium*, the ancestral area for most nodes was East Eurasia and North America, indicating subsequent dispersal from either East Eurasia or North America to West Eurasia. There is no obvious geographical barrier between East and West Eurasia, and many plants, such as *Aesculus* [24], *Sibbaldia* [25] and *Oxyria digyna* [26], spread from Asia to Europe through this route. Alternatively, an important route for plant migration between North America and Europe is the North Atlantic Land Bridge (NALB) [27, 28]. The lifespan of the NALB has been debated, with previous studies suggesting that the NALB was an effective dispersal route between North America and Europe until the Eocene or possibly early Miocene [29–34], but a recent study suggested that this land bridge did not close until the late Miocene (8–10 Ma) [35]. Therefore, the subgenus *Xanthosparganium,* in which most nodes differentiated during the Miocene, could have plausibly spread from North America to Europe through the NALB. Another possible dispersal route was from East Eurasia to the Indo-Pacific and Australia. During the late Oligocene, the Wallace District was uplifted by the collision of the Sunda Shelf with the Sahul Shelf, which led to the formation of a land bridge between Asia and Australia in the Miocene [36, 37]. The two Asia/Australia species *S. fallax* and *S. subglobosum*, both of which diverged in the middle Miocene, could have dispersed from Asia to Australia via the land bridge. Alternatively, long distance endozoochoric dispersal could have introduced *Sparganium* to more distant locations such as Australia, because the seeds of *Sparganium* plants can be eaten by migratory birds and spread over long distances [1, 38, 39]. A vicariance event suggested by BioGeoBEARS analysis occurs at the node of the Eurasian *S. gramineum* and the North American *S. fluctuans* with their divergence at 6.79 Ma (Figs. 3, 4, Table S2) involving the Beringian Land Bridge (BLB). The BLB served as an important route for the exchange of temperate plants between eastern Asia and western North America from the early Paleocene to Pliocene [40–42], and the final closure of the BLB occurred at 5.5–5.4 Ma [43]. Therefore, the ancestral area of East Eurasia + North America was likely sundered by the closing of the BLB, thus giving rise to *S. gramineum* and *S. fluctuans*.

Our time estimation (4.37 Ma) of the crown node of the subgenus *Sparganium* closely matches to the final closure of the BLB, thus indicating the crown diversification of the subgenus *Sparganium* associated with the vicariance event was likely invoked by the closure of the BLB. Within the subgenus *Sparganium*, other than the node leading to *S. erectum*/S*. erectum* subsp. *microcarpum*, the most likely ancestral area was North America. This subgenus likely dispersed from North America to Asia or Europe possibly via the NALB and BLB land bridges. The final closure of the BLB at 5.5–5.4 Ma [43] is far earlier than the relatively recent species divergence, with the earlier closure of the NALB, suggesting the spread from North America to Asia or Europe through long distance dispersal by birds. Both dispersal and vicariance events occurred in three out of five nodes within the subgenus *Sparganium* (Table S2), thus indicating that long distance dispersal and vicariance played an important role in the North American-European/Asian diversification of the subgenus *Sparganium*. Future phylogeographic investigations based on plants sampled from a wider geographical area could provide further insight into the biogeographical history of *Sparganium*.

## Conclusion

IN this study, we assembled 19 chloroplast genomes from *Sparganium* samples that each represented a distinct evolutionary lineage. The chloroplast genomes of *Sparganium* species have conserved genome structure, gene content, and gene order. Our phylogenomic analysis presented a well-resolved phylogeny of *Sparganium* species, although there remains some uncertainty surrounding taxonomic classification within the recently diversified subgenus *Sparganium*. We also reappraised the divergence time and historical biogeography of *Sparganium*: *Sparganium* diversified into two subgenera in the Oligocene. The subgenus *Xanthosparganium* began to diversify in the late Oligocen and then dispersed from eastern Eurasia and North America into western Eurasia and Australia. The subgenus *Sparganium* diversified in the late Pliocene and mostly expanded its range from North America into Eurasia. In summary, our study provides new insights into the chloroplast genome evolution, phylogeny, and biogeography of the genus *Sparganium*.

## Methods

### Plant sampling and DNA extraction

A total of 19 *Sparganium* samples comprising 15 putative species (including two samples of *S. eurycarpum,* one from Nova Scotia and one from Ontario, Canada) and three subspecies were collected (Table 1). Voucher specimens were kept in the herbaria of IBIW, Wuhan University and Trent University with specific voucher numbers (Table 1). Eugeny A. Belyakov and Xinwei Xu performed formal identification of the samples. Our species delimitations follow accepted names of *Sparganium* from Plants of the World Online (POWO), which incorporates the latest published taxonomy. The pictures of each species were presented in Figure S3. Total genomic DNA was extracted from silica-dried leaves using a DNA Secure Plant Kit (Tiangen Biotech, Beijing, China) following the manufacturer’s protocol.

### Genome skimming, chloroplast genome assembly and annotation

Library preparation and genomic sequencing on the Illumina Hiseq 2500 platform were conducted by Benagen (Benagen Inc., Wuhan, China). Approximately 10G paired-end reads (150 bp) were produced for each sample. The chloroplast genomes were assembled de novo using SPAdes v3.9.0 [44] after raw reads were trimmed and filtered using Fastp v0.20.0 [45] with default parameters. Gene annotation was conducted using Geseq [46] with the chloroplast genome of *Typha latifolia* (GU195652.1) [47] as a reference. The circular map of chloroplast genomes was created using OGDRAW v1.3.1 [48]. The 19 *Sparganium* chloroplast genomes were deposited in GenBank (see Table 1 for accession numbers). Two of them, *S. fallax* and *S. stoloniferum* subsp. *choui*, were reported in our two previous studies [49, 50].

### Comparative analysis of chloroplast genomes

The online program IRscope [51] was used to visualize the junction sites of the chloroplast genomes. Sequences of chloroplast genomes were aligned using MAFFT v7.221 [52]. The sequence and structural variations of *Sparganium* chloroplast genomes were identified using mVISTA [53] with the chloroplast genome of *T. latifolia* as a reference. Nucleotide diversity (Pi) was assessed using DnaSP v6.0 [54]. The sliding window method was used with a window length of 800 bp and step size of 200 bp.

### Phylogenetic analysis

The chloroplast genomes of *T. latifolia*, *T. orientalis* (MN602748.1) [55], *Ananas comosus* (KR336549.1) [56], and *Tillandsia usneoides* (KY293680) [57] were downloaded from GenBank as outgroups. The PCGs were extracted from each of the chloroplast genomes and used in the phylogenetic analyses. Sequences of PCGs were aligned using MAFFT v7.221 [52]. The best-fit model of nucleotide substitution was estimated by ModelFinder [58]. Maximum likelihood (ML) and Bayesian inference (BI) methods were used for phylogenetic inference. The ML analysis was performed using RAxML v8.2.12 [59] and 1000 repetitions were performed to summarize the ML bootstrap support. BI implemented in MrBayes v3.2.7 [60] was conducted using two independent runs of 10 million generations, and each run employed four Markov chains, with sampling at every 1,000 generations. Chain convergence was checked using Tracer v1.7.1 [61], and posterior probabilities (PP) were generated from trees after excluding a burn-in of the initial 25% of the trees.

### Molecular dating and ancestral area reconstruction

Two calibration points were used for divergence time estimation conducted in BEAST v1.7.4 [62]. One was the stem age of *Typha*—a minimum age of 70 Ma based on fossil evidence as used in previous studies [6, 63, 64]. The detailed setting was a lognormal prior with an offset of 70, a mean of 1.5, and a standard deviation of 0.5. The other was the stem age of Typhaceae that has a uniform distribution ranging from 90–105 Ma obtained from Givnish et al. [8] and used in a previous study [6]. Markov chain Monte Carlo (MCMC) analyses of 2 × 10^9^ generations were implemented, and every 1,000 generations were sampled. The initial 25% generations were discarded as burn-in, and the effective sample size (ESS) for the convergence of each parameter was checked using Tracer v1.7.1 [61].

Ancestral area reconstruction was conducted using the BioGeoBEARS package [65] implemented in RASP v4.0 [66]. Six geographical areas were defined based on the distribution of *Sparganium*: (A) North America, (B) Indo-Pacific, (C) West Eurasia, (D) East Eurasia, (E) Africa, and (F) Australia. The input trees for BioGeoBEARS analysis were obtained from BEAST analysis. The best-fit biogeographic model was determined according to the Akaike Information Criterion cumulative weight (AICc_wt).

## Supplementary Information

**Additional file 1:** **Figure S1.** Alignment of chloroplast genomes of* Sparganium* species. The chloroplast genome of *Typha latifolia* was used as a reference.

**Additional file 2:** **Figure S2.** Nucleotide diversity hotspot regions in chloroplast genomes of* Sparganium* species.

**Additional file 3:** **Figure S3.** Pictures of *Sparganium *species.

**Additional file 4:** **Table S1.** Sequence similarity among the chloroplast genomes of *Sparganium* species.

**Additional file 5:** **Table S2.** Ancestral area reconstruction using the BioGeoBEARS analysis based on the chronogram inferred using BEAST. Node number is indicated in Figure 4. Letter codes represent biogeographic regions: A - North America, B - Indo-Pacific, C - West Eurasia, D - East Eurasia, E - Africa, F - Australia. Supports for specific reconstructions are indicated by the values to the right of the letter code.

## Data Availability

All chloroplast genomes in this study are available from the National Center for Biotechnology Information (NCBI) (accession numbers: MW789349, MW810982- MW810984, MW817014-MW817019, MW829761-MW829768, and MW874418).
