# Supplementary material for: A reappraisal of the phylogeny and historical biogeography of Sparganium (Typhaceae) using complete chloroplast genomes
Source: BMC Plant Biol. 2022 Dec 15;22:588. doi: 10.1186/s12870-022-03981-3 (PMC9753266; doi:10.1186/s12870-022-03981-3)
Supplement: Supplementary file 1 — Additional file 1: Figure S1. Alignment of chloroplast genomes of Sparganium species. The chloroplast genome of Typha latifolia was used as a reference. [file 12870_2022_3981_MOESM1_ESM.pdf]

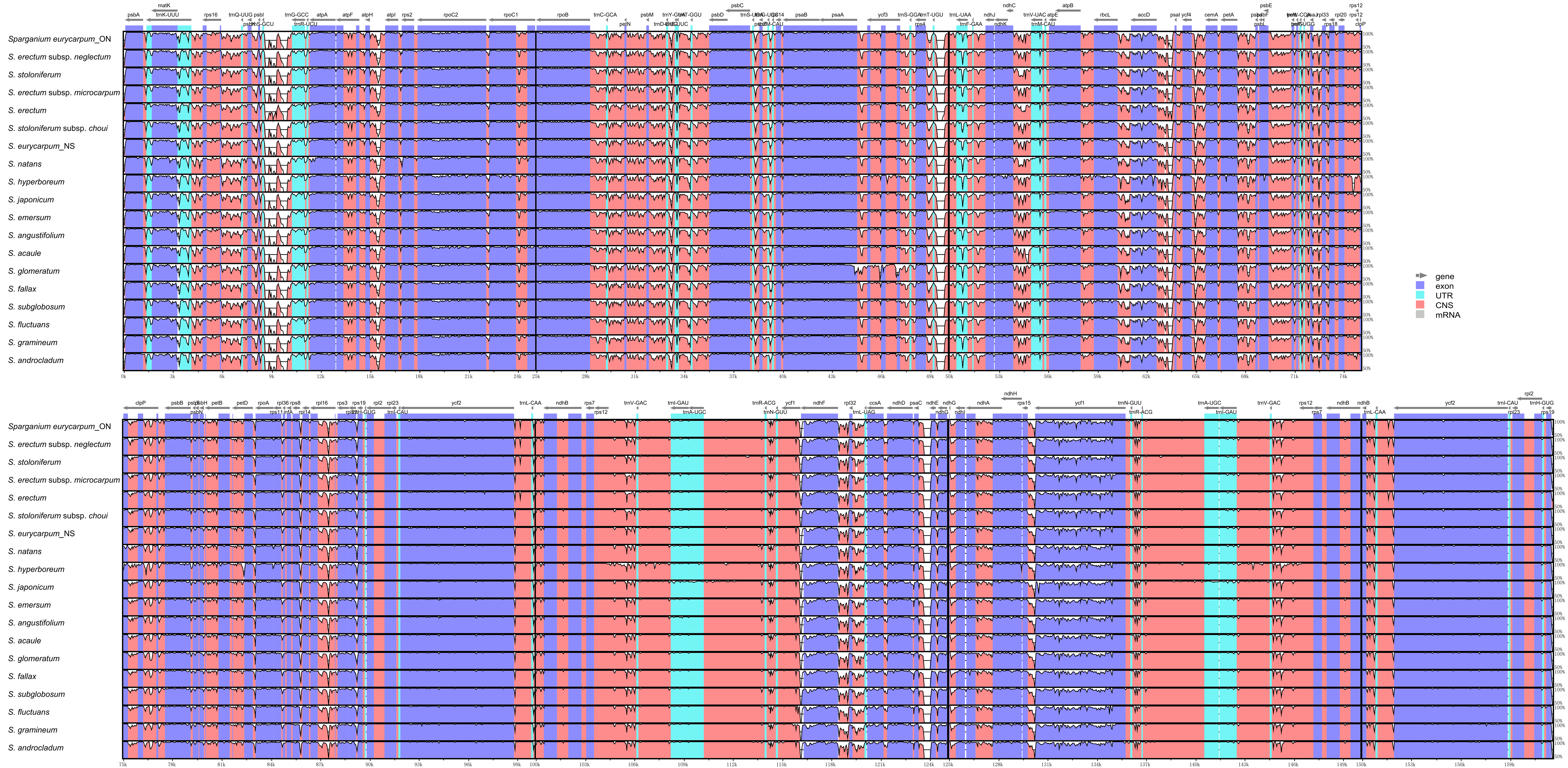

Figure S1. Alignment of chloroplast genomes of *Sparganium* species. The chloroplast genome of *Typha latifolia* was used as a reference.
