# Supplementary figures and images for: A reappraisal of the phylogeny and historical biogeography of Sparganium (Typhaceae) using complete chloroplast genomes
Source: BMC Plant Biol. 2022 Dec 15;22:588. doi: 10.1186/s12870-022-03981-3 (PMC9753266; doi:10.1186/s12870-022-03981-3)

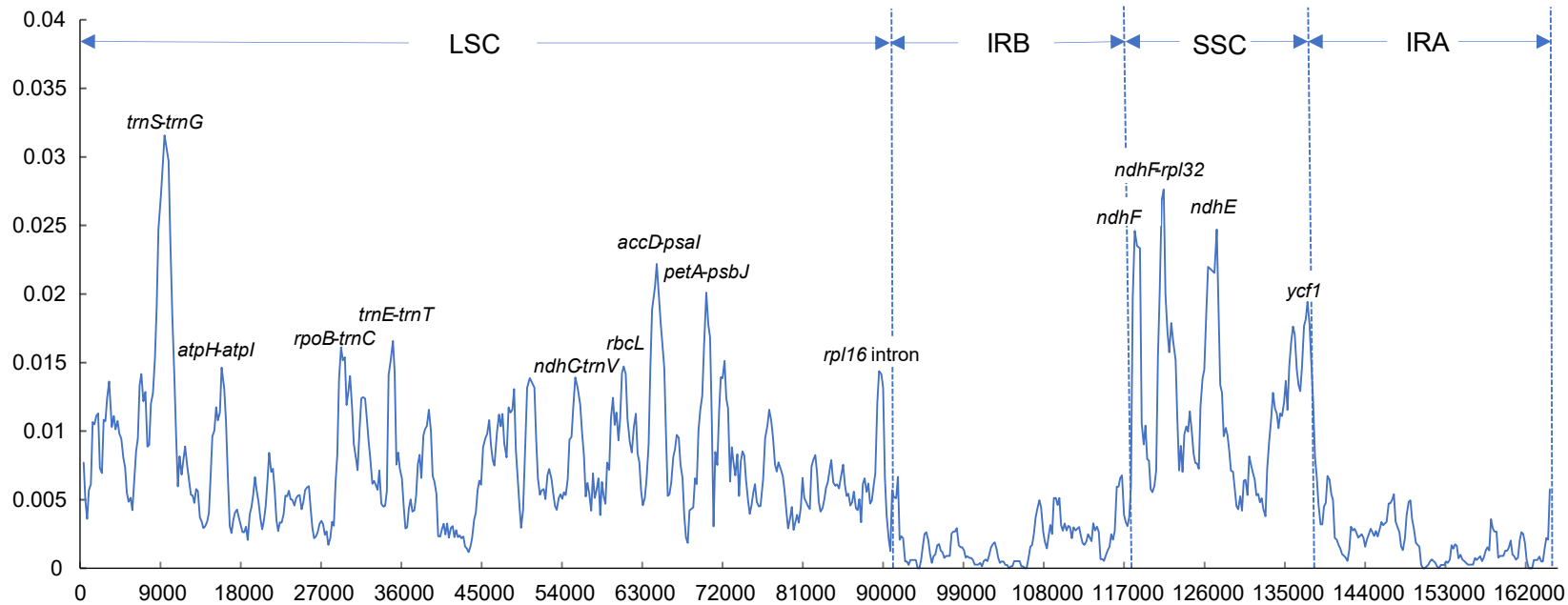

Figure S2. Nucleotide diversity hotspot regions in chloroplast genomes of *Sparganium* species.

Supplement: Supplementary file 2 — Additional file 2: Figure S2. Nucleotide diversity hotspot regions in chloroplast genomes of Sparganium species. [file 12870_2022_3981_MOESM2_ESM.pdf]
