# Supplementary material for: A reappraisal of the phylogeny and historical biogeography of Sparganium (Typhaceae) using complete chloroplast genomes
Source: BMC Plant Biol. 2022 Dec 15;22:588. doi: 10.1186/s12870-022-03981-3 (PMC9753266; doi:10.1186/s12870-022-03981-3)
Supplement: Supplementary file 3 — Additional file 3: Figure S3. Pictures of Sparganium species. [file 12870_2022_3981_MOESM3_ESM.pdf]

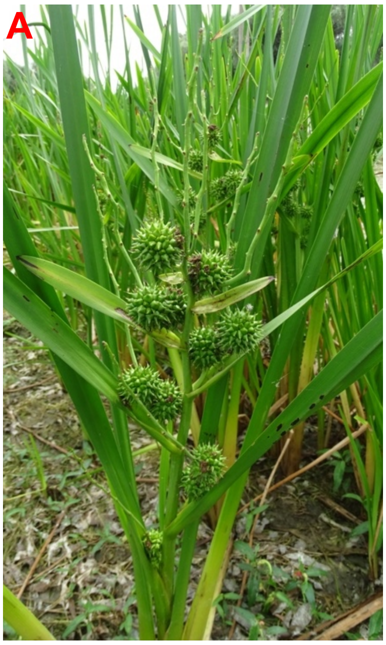

Sparganium erectum

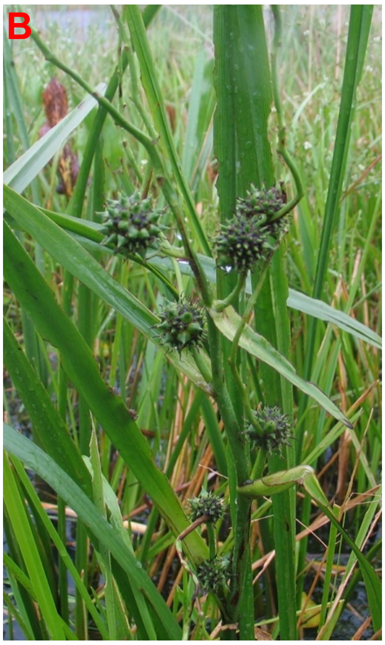

S. stoloniferum

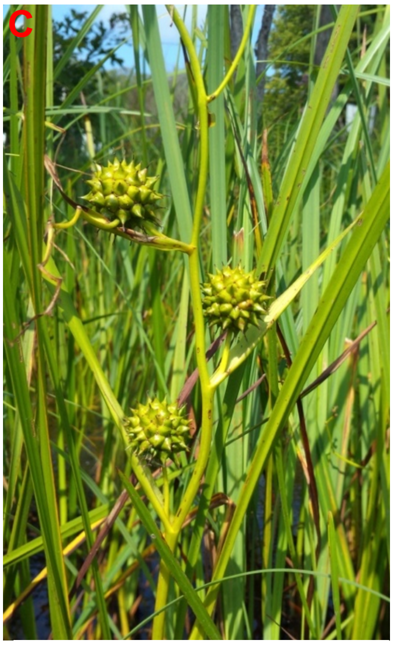

S. eurycarpum

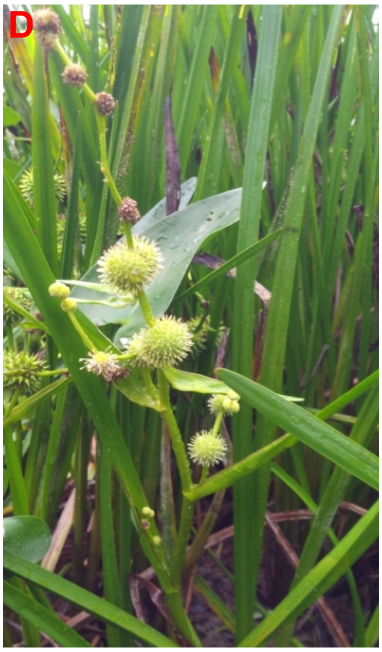

S. androcladum

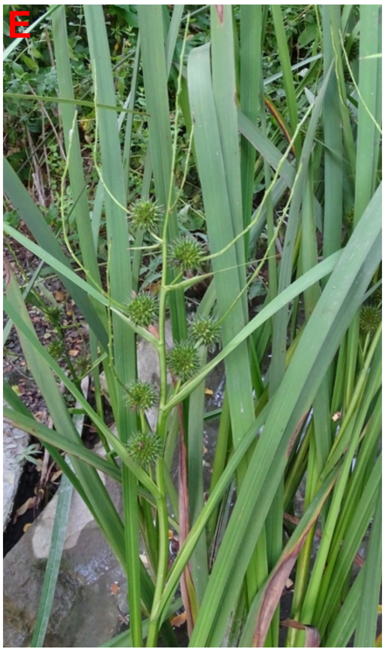

S. erectum subsp. *neglectum*

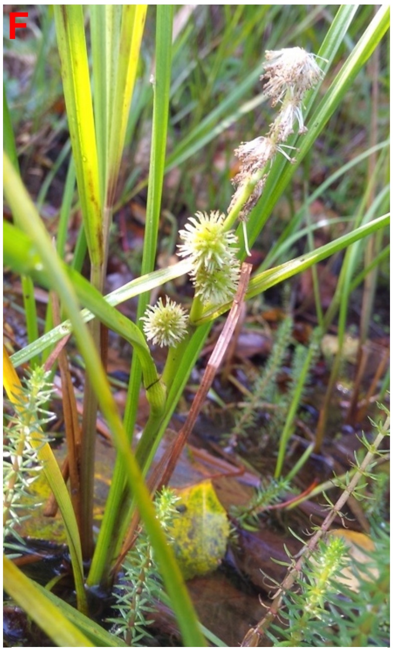

S. acaules

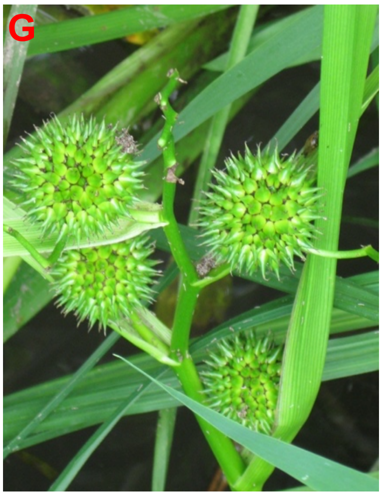

S. erectum subsp. *microcarpum*

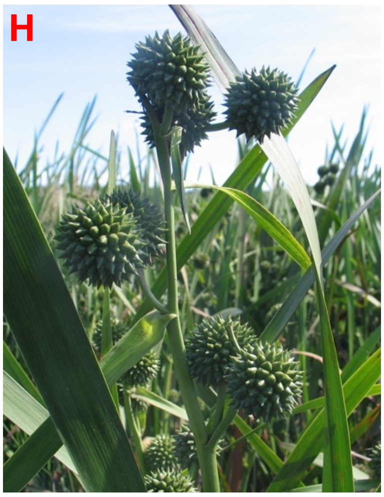

S. stoloniferum subsp. *choui*

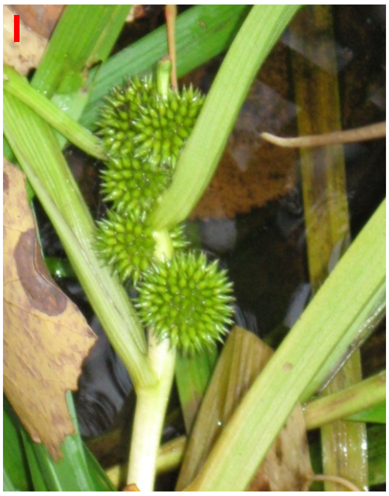

S. glomeratum

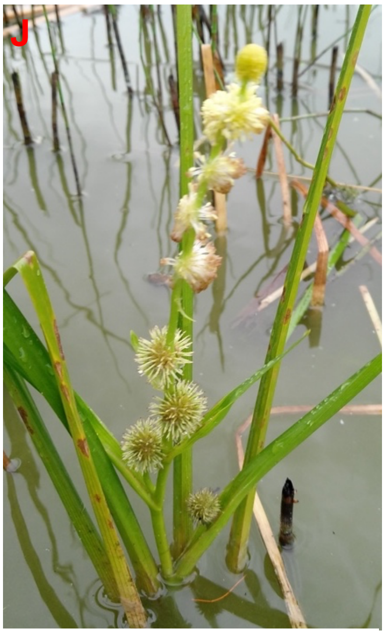

S. emersum

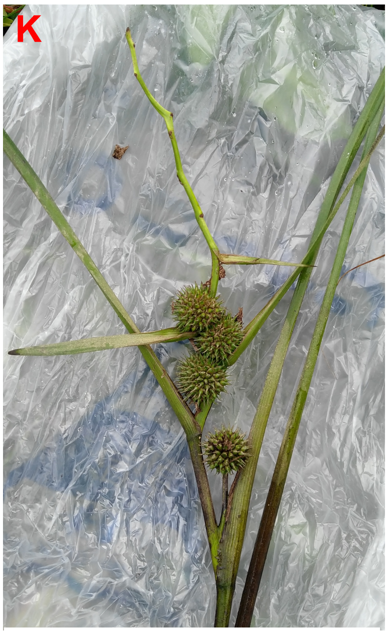

S. fallax

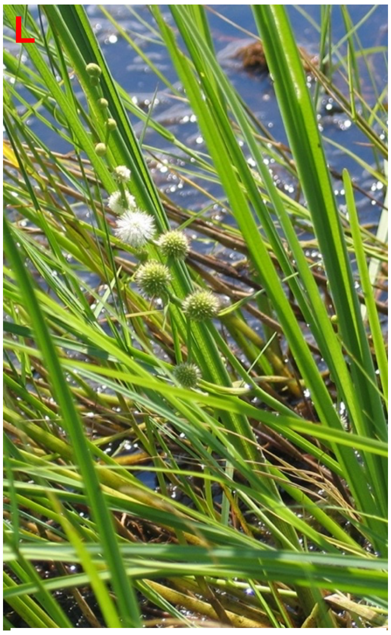

S. subglobosum

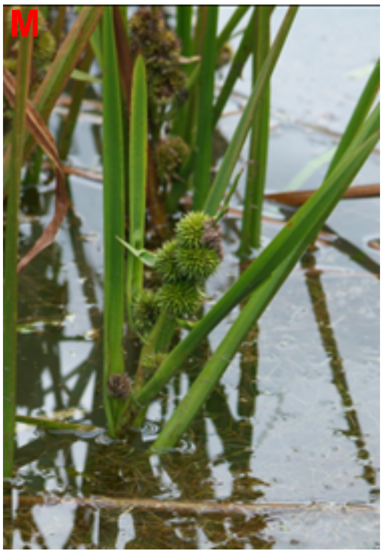

S. japonicum

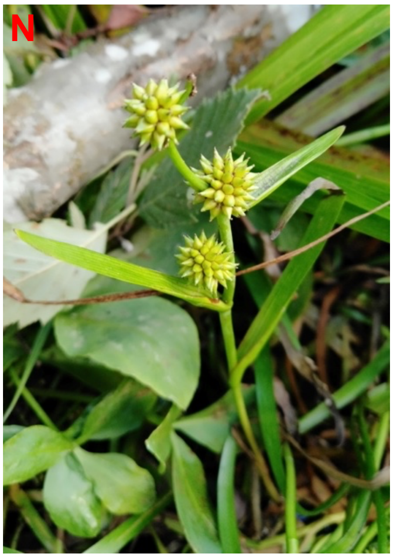

S. natans

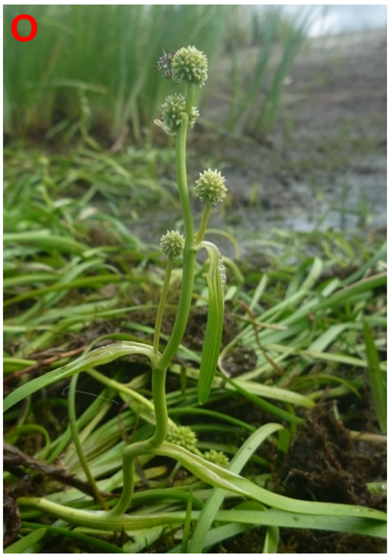

S. hyperboreum

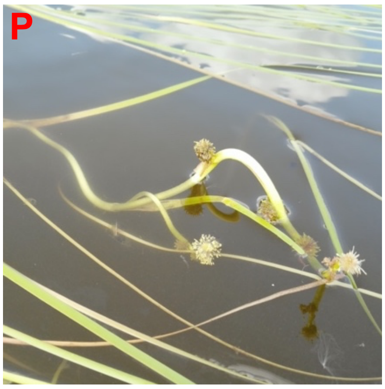

S. gramineum

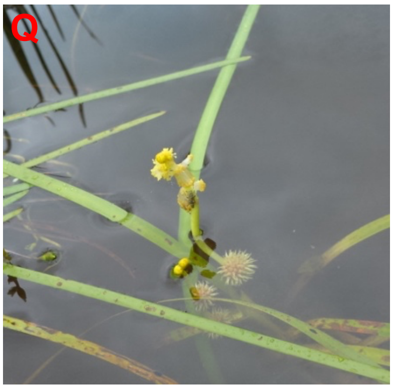

S. fluctuans

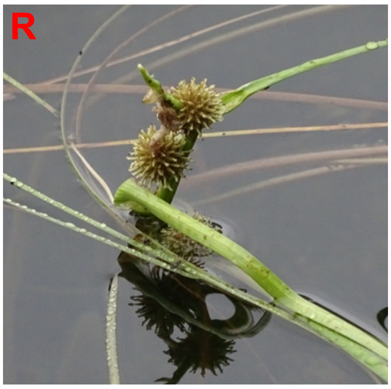

S. angustifolium

Figure S3. Pictures of *Sparganium* species. Pictures A, E, G, P, I and R were taken by E. A. Belyakov and A. G. Lapirov. Picture M was taken by D. Wang. Picture O was taken by S. A. Nikolaenko. All other pictures were taken by X. Xu.
