# Supplementary material for: A reappraisal of the phylogeny and historical biogeography of Sparganium (Typhaceae) using complete chloroplast genomes
Source: BMC Plant Biol. 2022 Dec 15;22:588. doi: 10.1186/s12870-022-03981-3 (PMC9753266; doi:10.1186/s12870-022-03981-3)
Supplement: Supplementary file 5 — Additional file 5: Table S2. Ancestral area reconstruction using the BioGeoBEARS analysis based on the chronogram inferred using BEAST. Node number is indicated in Figure 4. Letter codes represent biogeographic regions: A - North America, B - Indo-Pacific, C - West Eurasia, D - East Eurasia, E - Africa, F - Australia. Supports for specific reconstructions are indicated by the values to the right of the letter code. [file 12870_2022_3981_MOESM5_ESM.doc]

**Table S2** Ancestral area reconstruction using the BioGeoBEARS analysis based on the chronogram inferred using BEAST. Node number is indicated in Figure 4. Letter codes represent biogeographic regions: A - North America, B - Indo-Pacific, C – West Eurasia, D - East Eurasia, E - Africa, F - Australia. Supports for specific reconstructions are indicated by the values to the right of the letter code.

| Node | Ancestral areas | |  | Event | |  | RASP ROUTE |
| --- | --- | --- | --- | --- | --- | --- | --- |
|  | Area | Probability |  | Dispersal | Vicariance |  |  |
| 22 | ACD | 99.64 |  | 3 | 0 |  | ACD>ACD^A^C^D>ACD|ACD |
| 23 | AD/ACD/D | 96.21/2.04/1.74 |  | 2 | 0 |  | AD>AD^D>ACD^D>D|ACD |
| 24 | AD/D/ACD | 96.84/1.71/1.41 |  | 2 | 0 |  | AD>AD^D>ABD^D>BD|AD |
| 25 | AD/ACD | 98.22/1.65 |  | 2 | 0 |  | AD>AD^A>ACD^A>A|ACD |
| 26 | AD/ACD | 97.96/1.40 |  | 2 | 0 |  | AD>AD^A^D>AD|AD |
| 27 | AD/ACD | 97.94/1.40 |  | 3 | 0 |  | AD>AD^D>ABDF^D>BDF|AD |
| 28 | AD/ACD | 97.68/1.68 |  | 1 | 1 |  | AD>ACD>A|CD |
| 29 | AD/ACD | 98.26/1.45 |  | 2 | 0 |  | AD>AD^A^D>AD|AD |
| 30 | AD/ACD | 98.22/1.46 |  | 1 | 0 |  | AD>AD^A>A|AD |
| 31 | ACD/AD | 95.65/4.35 |  | 3 | 0 |  | ACD>ACD^A^C^D>ACD|ACD |
| 32 | AD/ACD | 87.9311.98 |  | 3 | 0 |  | AD>AD^A^D>ACD^A^D>ACD|AD |
| 33 | A/C/AC | 83.61/14.84/1.49 |  | 1 | 1 |  | A>AC>C|A |
| 34 | A/C/AC | 83.15/14.66/1.42 |  | 2 | 1 |  | A>ADF>DF|A |
| 35 | C/AC | 98.34/1.65 |  | 0 | 0 |  | C>C^C>C|C |
| 36 | A/C/AC | 83.12/14.66/1.53 |  | 1 | 1 |  | A>CA>C|A |
| 37 | A/AC/C | 91.50/5.00/2.64 |  | 0 | 0 |  | A>A^A>A|A |
| 38 | AD/ACD/CD | 91.16/5.04/2.61 |  | 0 | 1 |  | AD>D|A |
| 39 | AD/ACD | 88.09/11.47 |  | 2 | 0 |  | AD>AD^A^D>AD|AD |
| 40 | / | / |  | 8 | 1 |  | >^A^C^D>ABCDE^A^C^D>ABCD|ACDE |
| 41 | ACD/ACDE/ABCD | 86.97/8.91/4.04 |  | 9 | 0 |  | ACD>ACD^A^A^C^D^D^A^D>ABCDE^A^A^C^D^D^A^D  >ACDE|ABCD|AD |
